# Cytomegalovirus reactivation in the lower respiratory tract as an independent risk factor for mortality in critically Ill patients

**DOI:** 10.1186/s13054-025-05324-8

**Published:** 2025-05-02

**Authors:** Joong-Yub Kim, Chan Mi Lee, Yoon Hae Ahn, Hong Yeul Lee, Sang-Min Lee, Hyeon Jae Jo, Pyoeng Gyun Choe, Wan Beom Park, Chang Kyung Kang, Jinwoo Lee, Nam Joong Kim

**Affiliations:** 1 Division of Pulmonary and Critical Care Medicine, Department of Internal Medicine, Seoul National University Hospital, Seoul, Republic of Korea; 2 Department of Internal Medicine, Seoul National University Hospital, Seoul National University College of Medicine, Seoul, Republic of Korea; 3 Department of Critical Care Medicine, Seoul National University Hospital, Seoul, Republic of Korea; 4 Department of Microbiology, Harvard Medical School, Boston, MA USA

**Keywords:** Lower respiratory tract, CMV, ICU, Reactivation, Prognosis

## Abstract

**Background:**

The clinical significance of cytomegalovirus reactivation in the lower respiratory tract (LRT) of critically ill patients remains unclear. We aimed to investigate the association between cytomegalovirus reactivation detected in LRT and intensive care unit (ICU) prognosis.

**Methods:**

This study included critically ill patients admitted to a medical ICU at a tertiary referral center in South Korea between January 2021 and June 2023. Cytomegalovirus load in LRT samples collected via bronchoscopy was measured within 7 days of admission. Detection of cytomegalovirus DNA in LRT was defined as reactivation. Associations between cytomegalovirus reactivation and ICU, in-hospital, 30-day, and 90-day mortality were assessed using multivariable Fine-Gray model adjusted for major clinical factors.

**Results:**

Of the 322 patients (median age 68 years, 66.8% male), 145 (45%) had cytomegalovirus reactivation in the LRT. Cytomegalovirus reactivation was independently associated with increased ICU (adjusted subdistribution hazard ratio [aSHR], 2.28; 95% confidence interval [CI], 1.46–3.56), in-hospital (aSHR, 2.00; 95% CI, 1.44–2.78), 30-day (aSHR, 2.11; 95% CI, 1.42–3.13), and 90-day mortality (aSHR, 2.05; 95% CI, 1.45–2.88). Anti-cytomegalovirus therapy was significantly associated with reduced ICU mortality in patients with radiologic findings suggestive of cytomegalovirus pneumonia (*P* for interaction = 0.001), but was linked to increased mortality in patients with positive bacterial cultures (*P* for interaction = 0.002).

**Conclusion:**

Cytomegalovirus reactivation in the LRT is associated with poor outcomes in critically ill patients. Anti-cytomegalovirus therapy was not associated with overall survival outcomes; however, the subgroup with radiologic findings of cytomegalovirus pneumonia suggested benefits, while the subgroup with bacterial co-infections suggested harmful effects. Randomized controlled trials are needed.

**Supplementary Information:**

The online version contains supplementary material available at 10.1186/s13054-025-05324-8.

## Introduction

Cytomegalovirus (CMV) is a ubiquitous beta-herpesvirus that infects humans worldwide [1]. CMV is acquired early in life through exposure to infected body fluids, causing conditions ranging from asymptomatic infection to infectious mononucleosis, CMV syndrome, and CMV end-organ disease, followed by lifelong latency [2, 3]. CMV seropositivity prevalence in adults varies geographically, with South Korea showing rates approaching 95% of the general population [4–6]. CMV reactivation, where the virus resumes replication from the latent state, frequently occurs in critical illnesses, affecting 20% to 40% of cases [7, 8]. This reactivation is associated with worse clinical outcomes and increased healthcare resource use [9–11]. While previous research has focused on the link between plasma CMV viral load and clinical outcomes, studies on CMV reactivation in the lower respiratory tract (LRT) and its clinical significance are limited [12].

LRT specimen collection via bronchoscopy is a common procedure in patients with acute respiratory failure, providing crucial diagnostic information, particularly for confirming or ruling out respiratory infections [13, 14]. One of the diagnostic tests performed on LRT samples include real-time polymerase chain reaction (PCR) to detect CMV deoxyribonucleic acid (DNA). However, the clinical implications of detecting CMV DNA in the LRT specimen of critically ill patients, especially those who are not highly suspicious for CMV pneumonia, remain unclear [12].

The primary objective of this study was to examine the association between CMV reactivation, detected in LRT specimen, and clinical outcomes in critically ill patients. Additionally, we aimed to identify specific patient subgroups that may benefit from CMV-targeted antiviral therapy.

## Methods

### Ethics statement

This study was approved by the Institutional Review Board of Seoul National University Hospital (approval number: H-2308-071-1457) with a waiver for obtaining informed consent owing to the retrospective nature of the study design. This study was conducted according to the principles of the Declaration of Helsinki.

### Study population

We analysed critically ill patients aged ≥ 19 years who were admitted to the medical intensive care unit (ICU) between January 1, 2021 and June 30, 2023 at a tertiary referral centre in South Korea and had undergone bronchoscopic LRT specimen collection within 7 days of ICU admission. In our medical ICU, flexible fiberoptic bronchoscopy is performed to obtain LRT specimen in patients with acute respiratory failure when there is clinical suspicion of a LRT infection and noninvasive diagnostic tests are inconclusive. The decision is made collaboratively by the treating intensivist and the pulmonary team, considering clinical severity, radiologic findings, and the necessity for microbiological confirmation. Given that bronchoscopy is a semi-invasive procedure, performing a comprehensive panel of tests on the obtained specimen concurrently, including CMV viral load testing, is part of the standard clinical care protocol to capture a broad range of diagnostic clues that could aid in elucidating the cause of respiratory failure. CMV viral load was quantified using Abbott RealTime CMV PCR assay (Abbott, Illinois, USA) according to the manufacturer’s instructions and reported in IU/mL, standardized to the 1st WHO International Standard for human CMV for nucleic acid amplification tests (NIBSC 09/162) [15]. Patients without CMV viral load testing from LRT specimen, those who had initiated antiviral agents against CMV before the bronchoscopic procedure, and those who stayed in the ICU for < 24 h were excluded.

### Data collection and outcomes

We retrospectively collected demographic, clinical, and laboratory data including age, sex, body mass index (BMI), disease severity scores such as APACHE II and SOFA scores, underlying comorbidities, Charlson comorbidity index, initial PaO2/FiO2 ratio at ICU admission, lactate levels, C-reactive protein (CRP), plasma CMV viral load and antigen positivity within 2 weeks before ICU admission or 1 week after admission, as well as the initiation of mechanical ventilation and continuous kidney replacement therapy (CKRT). Ventilator-free days (VFD) during the first 28 days were defined as the number of days a patient was alive and not on mechanical ventilation. Patients who died within this period were assigned 0 VFD [16]. We also gathered data on lymphopenia at the time of LRT specimen collection, a marker of acute immune suppression in critically ill patients [17, 18], defined as an absolute lymphocyte count < 1,000 cells/µL in blood [19].

Radiological findings suggestive of CMV pneumonia were defined as the presence of diffuse bilateral ground-glass opacities, a characteristic feature of CMV pneumonia [20], as observed on computed tomography performed within 2 weeks of the bronchoscopic procedure. In the LRT specimen analysis, we collected data on CMV viral load, bacterial and fungal culture results, *Pneumocystis jirovecii* PCR, and respiratory viral PCR results. We also collected data on the use of corticosteroids or other immunosuppressants, including anti-neoplastic agents, disease-modifying antirheumatic drugs, calcineurin inhibitors, mTOR inhibitors, and antiproliferative drugs. Use of these drugs was defined as corticosteroids equivalent to ≥ 10 mg of prednisolone [21] or any other immunosuppressants administered within four weeks prior to ICU admission. We also gathered data on the administration of antiviral agents against CMV, such as ganciclovir, valganciclovir, cidofovir, and foscarnet, and their initiation dates following ICU admissions. Antiviral therapy was initiated at the discretion of the attending physicians, guided by the patient’s underlying disease, the severity of critical illness, and radiographic and laboratory findings. Intravenous ganciclovir was the primary antiviral agent used, typically administered for 2 to 4 weeks at a therapeutic dose. The primary outcome was ICU mortality. The secondary outcomes were the in-hospital, 30-day, and 90-day mortality rates.

### Statistical analysis

Clinical characteristics at ICU admission were compared between patients with positive LRT CMV results, defined as viral loads ≥ 34.5 IU/mL, the lower limit of quantification in our laboratory, and those with negative results. Variables are summarized as frequency with proportion or median with interquartile range (IQR). Pearson’s chi-squared or Fisher’s exact test and Mann–Whitney *U* test were used to compare categorical and non-parametric continuous variables, respectively. We assessed the correlation between plasma CMV DNA positivity and LRT CMV results, and calculated the diagnostic performance of plasma CMV DNA in predicting positive LRT CMV, including sensitivity, specificity, and predictive values.

For survival analysis, we utilized the cumulative incidence function and the Fine–Gray subdistribution hazard model to account for competing risks such as ICU or hospital discharge when evaluating the association between LRT CMV positivity and outcomes. Given the potential for time differences, we designated the date of LRT specimen collection, rather than ICU admission, as the index date. For the multivariable analysis, we selected important demographic factors along with variables related to disease severity and prognosis. Additionally, potential confounders that showed significant differences at *P* < 0.1 between the CMV-negative and positive groups were included. To address potential false negativity due to low instilled volume in patients who underwent bronchial washing, a sensitivity analysis was performed by isolating those with LRT samples collected via bronchial washing.

Finally, we conducted subgroup analyses to identify the subgroups for which antiviral agents against CMV may be beneficial. Because the timing of the initiation of antiviral agents varies individually over time, we applied a time-varying analysis to avoid immortal time bias [22, 23]. Time intervals were constructed from the date of bronchoscopy to antiviral treatment initiation and from treatment initiation to ICU discharge or death. To evaluate whether the effect of antiviral treatment on outcomes varies significantly between subgroups, we reported the *P* for interaction. The results of the subgroup analyses were summarized and presented in a forest plot. Statistical significance was set at *P* < 0.05. All analyses were conducted using the R software version 4.4.1 (R Foundation for Statistical Computing, Vienna, Austria).

## Results

### Study population

**Fig. 1:**
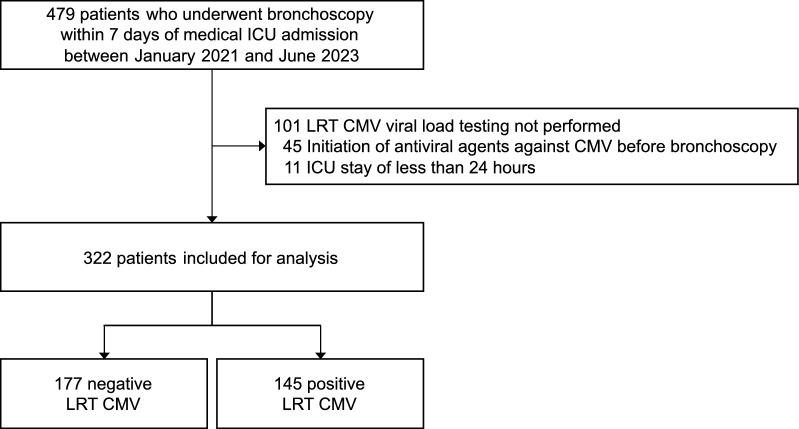
Flow diagram. LRT, lower respiratory tract; CMV, cytomegalovirus; ICU, intensive care unit

Of the 479 patients who underwent LRT sample collection via bronchoscopy within 7 days of medical ICU admission, 322 patients (median age, 68 years [IQR, 59–75 years]; 66.8% men) were included for the analysis. Figure 1 details the number of exclusions and their reasons. In total, 145 patients (45.0%) tested positive for LRT CMV (viral load ≥ 34.5 IU/mL), whereas 177 (55.0%) tested negative (Table 1). The median time to LRT collection from ICU admission was 1 day (IQR, 0–2 days), with no significant difference between the groups (*P* = 0.716). The median LRT CMV viral load among positive patients was 1,390 IU/mL (IQR, 236–13,300 IU/mL).

**Table 1:** Clinical characteristics of patients stratified by LRT CMV positivity

Characteristics	Study cohort (*n* = 322)	negative LRT CMV (*n* = 177)	positive LRT CMV (*n* = 145)	*P*
Time to LRT sample collection from ICU admission, days	1 (0–2)	1 (0–2)	1 (0–2)	0.716
Male sex	215 (66.8)	112 (63.3)	103 (71.0)	0.177
Age	68 (59–75)	67 (58–75)	69 (62–75)	0.103
Body mass index, kg/m^2^	21.2 (18.7–24.1)	22.0 (19.3–24.9)	20.6 (18.0–23.3)	0.001
Disease severity				
APACHE II score	19 (15–24)	18 (14–23)	20 (15–25)	0.017
SOFA score	8 (5–11)	8 (5–11)	8 (6–11)	0.869
Septic shock status	230 (71.4)	124 (70.1)	106 (73.1)	0.633
Comorbidities				
Solid organ malignancy	100 (31.1)	56 (31.6)	44 (30.3)	0.898
Hematologic malignancy	73 (22.7)	47 (26.6)	26 (17.9)	0.088
Solid organ transplantation	18 (5.6)	9 (5.1)	9 (6.2)	0.848
Hematopoietic stem cell transplantation	16 (5.0)	12 (6.8)	4 (2.8)	0.163
Diabetes mellitus	83 (25.8)	52 (29.4)	31 (21.4)	0.132
Chronic kidney disease	40 (12.4)	20 (11.3)	20 (13.8)	0.613
Congestive heart failure	19 (5.9)	8 (4.5)	11 (7.6)	0.355
Chronic obstructive pulmonary disease	14 (4.3)	4 (2.3)	10 (6.9)	0.079
Connective tissue disease	33 (10.2)	10 (5.6)	23 (15.9)	0.005
SARS-CoV-2	9 (2.8)	6 (4.4)	3 (2.8)	0.739
Charlson comorbidity index	5 (3–7)	4 (3–7)	5 (3–6)	0.234
Primary reasons for ICU admissions (multiple reasons allowed)				
Respiratory failure	311 (96.6)	171 (96.6)	140 (96.6)	> 0.999
Septic condition	238 (73.9)	140 (79.1)	98 (67.6)	0.027
Renal failure	57 (17.7)	30 (16.9)	27 (18.6)	0.807
Cardiovascular complications	36 (11.2)	25 (14.1)	11 (7.6)	0.094
Cardiac arrest	12 (3.7)	6 (3.4)	6 (4.1)	0.955
Liver failure	8 (2.5)	6 (3.4)	2 (1.4)	0.428
Others	25 (7.8)	13 (7.3)	12 (8.3)	0.919
Medications within 4 weeks prior to ICU admission				
Corticosteroid use	75 (23.3)	29 (16.4)	46 (31.7)	0.002
Other immunosuppressant use	142 (44.1)	75 (42.4)	67 (46.2)	0.564
Laboratory results at ICU admission				
Lactate, mmol/L	1.8 (1.2–2.9)	1.8 (1.3–2.9)	1.7 (1.2–2.9)	0.357
C-reactive protein, mg/dL	11.1 (4.7–20.6)	11.8 (4.9–22.2)	10.5 (4.5–19.2)	0.165
PaO2/FiO2 ratio (mmHg)	186 (127–275)	173 (126–250)	197 (132–297)	0.120
PaO2/FiO2 ≤ 100	142 (44.1)	71 (40.1)	71 (49.0)	
100 < PaO2/FiO2 ≤ 200	141 (43.8)	84 (47.5)	57 (39.3)	
200 < PaO2/FiO2 ≤ 300	39 (12.1)	22 (12.4)	17 (11.7)	
Lymphopenia at LRT sample collection	261 (81.1)	139 (78.5)	122 (84.1)	0.257
LRT CMV viral load, IU/mL	0 (0–1040)	0 (0–0)	1390 (236–13,300)	< 0.001
Plasma CMV positivity (≥ 34.5 IU/mL) (*n* = 291)	104 (35.7)	17 (10.3)	87 (69.0)	< 0.001
Plasma viral load among positive patients, IU/mL	519 (114–3775)	91 (58–138)	972 (186–5575)	< 0.001
Plasma CMV Ag positivity (*n* = 298)	51 (17.1)	3 (1.8)	48 (36.1)	< 0.001
Radiological findings suggestive of CMV pneumonia (*n* = 275)	179 (65.1)	98 (62.8)	81 (68.1)	0.437
LRT sample collection procedure				0.852
Bronchoalveolar lavage	105 (32.6)	59 (33.3)	46 (31.7)	
Bronchial washing	217 (67.4)	118 (66.7)	99 (68.3)	
Clinical outcomes				
MV application during study period	309 (96.0)	172 (97.2)	137 (94.5)	0.349
Time to MV from ICU admissions, days	0 (0–0)	0 (0–0)	0 (0–0)	0.286
Time to bronchoscopy from MV, days	1 (0–2)	1 (0–2)	1 (0–2)	0.930
Ventilator-free days during the first 28 days	0 (0–21)	8 (0–22)	0 (0–18)	0.006
CKRT	91 (28.3)	50 (28.2)	41 (28.3)	> 0.999
ICU mortality	99 (30.7)	42 (23.7)	57 (39.3)	0.004
In-hospital mortality	173 (53.7)	81 (45.8)	92 (63.4)	0.002
30-day mortality (*n* = 315)	122 (38.7)	54 (31.0)	68 (48.2)	0.003
90-day mortality (*n* = 300)	161 (53.7)	73 (44.2)	88 (65.2)	< 0.001
Anti-CMV agent use	40 (12.4)	7 (4.0)	33 (22.8)	< 0.001
Anti-CMV treatment duration, days	17 (6–27)	17 (8–41)	16 (7–25)	0.831

APACHE, Acute Physiology and Chronic Health Evaluation; LRT, lower respiratory tract; CMV, cytomegalovirus; ICU, intensive care unit; SOFA, Sequential Organ Failure Assessment; SARS-CoV-2, severe acute respiratory syndrome coronavirus 2; MV, mechanical ventilation; CKRT, continuous kidney replacement therapy. “*n*” represents the number of patients tested or with available data

### Clinical characteristics stratified by LRT CMV positivity

Patients with positive LRT CMV had a lower BMI (median, 20.6 *vs.* 22.0 kg/m^2^, *P* = 0.001), a higher APACHE II score (median, 20 *vs.* 18, *P* = 0.017), a greater prevalence of connective tissue disease (15.9% *vs.* 5.6%, *P* = 0.005), and a higher rate of corticosteroid use prior to ICU admission (31.7% *vs.* 16.4%, *P* = 0.002) than those with negative LRT CMV (Table 1). Patients with positive LRT CMV were also more likely to have positive *Aspergillus* antigen (38.6% *vs.* 21.8%, *P* = 0.002) in the LRT specimen (Table 2). Plasma CMV DNA positivity was observed in 69.0% of patients with positive LRT CMV results compared to 10.3% of those with negative LRT CMV results (*P* < 0.001, Table 1). When plasma CMV DNA was positive, its ability to predict positive LRT CMV (sensitivity) was 69.0%, specificity was 89.7%, negative predictive value was 79.1%, positive predictive value was 83.7%, and diagnostic accuracy was 80.8%. Other clinical factors, including age, sex, SOFA score, septic shock status, and comorbidities such as hematologic malignancy, were comparable between the two groups.

**Table 2:** LRT specimen test results by LRT CMV positivity

Pathogens	Study cohort (*n* = 322)	LRT CMV negative (*n* = 177)	LRT CMV positive (*n* = 145)	*P*
Bacterial culture positivity	115 (35.7)	55 (31.1)	60 (41.4)	0.071
Gram-negative bacteria	50 (15.5)	23 (13.0)	27 (18.6)	0.218
*Pseudomonas spp.*	8 (2.5)	3 (1.7)	5 (3.4)	0.518
*Acinetobacter spp.*	14 (4.3)	6 (3.4)	8 (5.5)	0.511
*Klebsiella spp.*	10 (3.1)	4 (2.3)	6 (4.1)	0.520
*E. coli spp.*	4 (1.2)	1 (0.6)	3 (2.1)	0.480
Gram-positive bacteria	72 (22.4)	34 (19.2)	38 (26.2)	0.172
*S. aureus*	10 (3.1)	6 (3.4)	4 (2.8)	0.998
*Streptococcus spp.*	7 (2.2)	4 (2.3)	3 (2.1)	> 0.999
*S. pneumoniae*	1 (0.3)	1 (0.6)	0 (0.0)	> 0.999
*Enterococcus spp.*	17 (5.3)	6 (3.4)	11 (7.6)	0.154
*Corynebacterium*	22 (6.8)	8 (4.5)	14 (9.7)	0.111
Coagulase-negative staphylococci	13 (4.0)	9 (5.1)	4 (2.8)	0.441
Fungi				
*Aspergillus spp.* positivity (*n* = 320)	94 (29.4)	39 (22.2)	55 (38.2)	0.003
By culture (*n* = 314)	12 (3.8)	3 (1.7)	9 (6.3)	0.069
By antigen test (Galactomannan) (*n* = 314)	92 (29.3)	38 (21.8)	54 (38.6)	0.002
*Pneumocystis jirovecii* PCR positive (*n* = 314)	56 (17.8)	26 (14.9)	30 (21.4)	0.179
Without bacterial or fungal pathogen identification (*n* = 320)	151 (47.2)	94 (53.4)	57 (39.6)	0.019
Respiratory virus				
Virus PCR^*^ positivity (*n* = 262)	11 (4.2)	6 (4.3)	5 (4.1)	> 0.999
HSV culture positivity (*n* = 202)	13 (6.4)	4 (3.6)	9 (9.9)	0.128

LRT, lower respiratory tract; CMV, cytomegalovirus; PCR, polymerase chain reaction; HSV, herpes simplex virus. “*n*” represents the number of patients who underwent the respective testing. ^*^The ‘virus PCR’ detects respiratory viruses including adenovirus, bocavirus, coronavirus (229E, NL63, OC43), enterovirus, influenza A and B viruses, metapneumovirus, parainfluenza virus (type 1, 2, 3, 4), respiratory syncytial virus (A and B), and rhinovirus (A/B/C). Since this test does not include HSV, HSV culture was done separately

### Association between LRT CMV positivity and clinical outcomes

Patients with positive LRT CMV showed no difference from those with negative results regarding mechanical ventilation application and CKRT during the study period (Table 1). However, the LRT CMV positive group had significantly fewer VFD days during the first 28 days.

Over a median ICU stay of 10 days (IQR, 6–16 days), 99 patients (30.7%) died. The in-hospital, 30-day, and 90-day mortality rates were 53.7%, 38.7%, and 53.7%, respectively. Patients with positive LRT CMV results had significantly higher rates of ICU mortality (39.3% *vs.* 23.7%, *P* = 0.004), in-hospital mortality (63.4% *vs.* 45.8%, *P* = 0.002), 30-day mortality (48.2% *vs.* 31.0%, *P* = 0.003), and 90-day mortality (65.2% *vs.* 44.2%, *P* < 0.001) than those with negative LRT CMV results (Table 1).

**Fig. 2:**
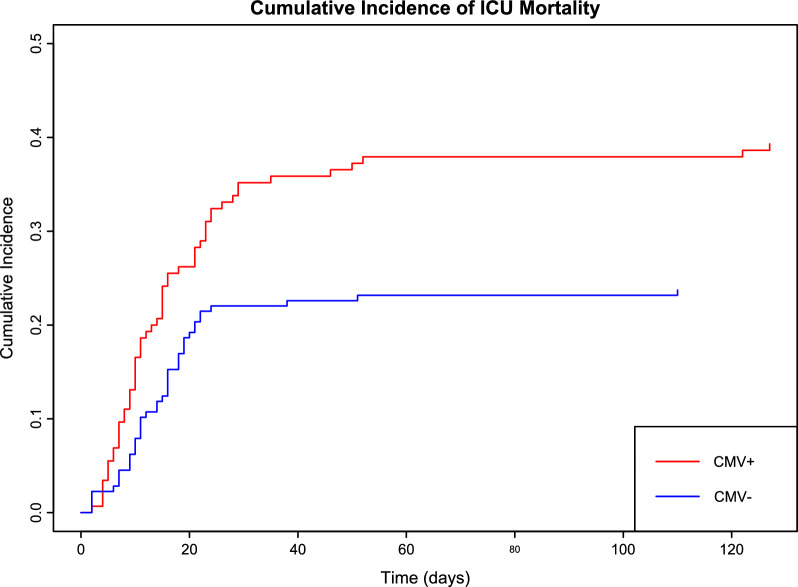
Cumulative incidence of ICU mortality by LRT CMV positivity. LRT, lower respiratory tract; CMV, cytomegalovirus; ICU, intensive care unit. Cumulative incidence function plot for ICU mortality, stratified by LRT CMV positivity. The plot depicts ICU mortality trends over time, with CMV-positive cases represented in red and CMV-negative cases in blue. Statistical significance of the differences between groups was assessed using Gray’s test

The cumulative incidence curve, stratified by LRT CMV positivity, showed significant divergence in ICU survival rates (*P* = 0.005; Fig. 2). Univariable Fine–Gray analysis demonstrated LRT CMV positivity was associated with ICU mortality (subdistribution hazard ratio [SHR], 1.82, 95% confidence interval [CI], 1.23–2.70, *P* = 0.003; Table 3 and Supplementary Table 1). Furthermore, multivariable analyses adjusted for sex, BMI, APACHE II score, hematologic malignancy, initial lactate level, lymphopenia, corticosteroid use, and LRT *Aspergillus* positivity revealed that positive LRT CMV was independently associated with increased ICU, in-hospital, 30-day, and 90-day mortality rates (Table 3).

**Table 3:** Association between LRT CMV positivity and mortality

Clinical outcome	SHR (95% CI)	*P*	adjusted SHR (95% CI)	*P*
ICU mortality	1.82 (1.23–2.70)	0.003	2.28 (1.46–3.56)	< 0.001
In-hospital mortality	1.67 (1.24–2.24)	< 0.001	2.00 (1.44–2.78)	< 0.001
30-day mortality	1.77 (1.25–2.50)	0.001	2.11 (1.42–3.13)	< 0.001
90-day mortality	1.70 (1.25–2.32)	< 0.001	2.05 (1.45–2.88)	< 0.001

LRT, lower respiratory tract; CMV, cytomegalovirus; SHR; subdistribution hazard ratio; CI, confidence interval; ICU, intensive care unit. Multivariable analysis using Fine–Gray model adjusted for sex, body mass index, APACHE II score, hematologic malignancy, initial lactate levels, lymphopenia, corticosteroid use, and *Aspergillus* positivity in LRT specimen was performed

In our cohort, 105 (32.6%) and 217 (67.4%) patients had samples obtained via bronchoalveolar lavage and bronchial washing, respectively. No significant differences in LRT CMV positivity was observed based on the sampling method (Table 1). A sensitivity analysis of patients who underwent bronchial washing, which may be prone to false negatives due to low instill volumes, yielded consistent results (Supplementary Table 2).

### Subgroup analyses regarding the use of antiviral agents against CMV

**Fig. 3:**
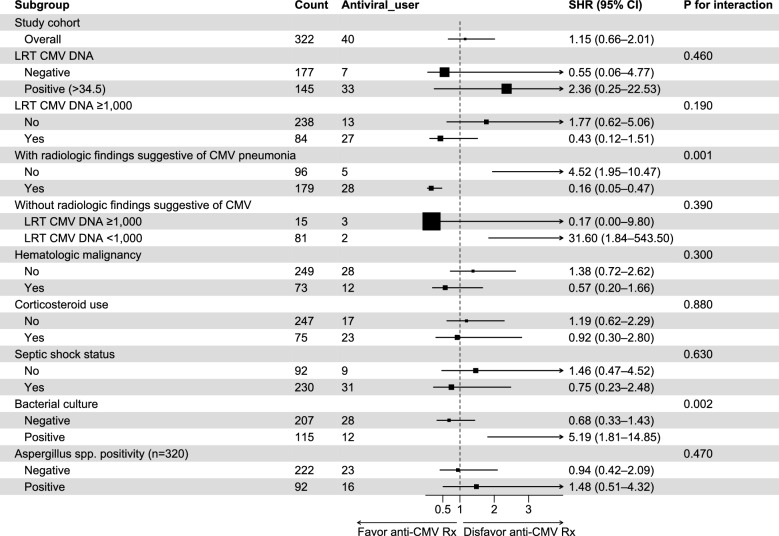
Forest plot illustrating the subdistribution hazard ratios of anti-CMV therapy on ICU mortality across various subgroups. LRT, lower respiratory tract; CI, confidence interval; CMV, cytomegalovirus; SHR, subdistribution hazard ratio; ICU, intensive care unit. Each subgroup displays the count and number of antiviral users, along with SHRs and 95% CI. Lines to the left favor anti-CMV therapy, while those to the right disfavor it. Larger squares indicate greater weight in the analysis. The *P* for interaction assess whether the effect of anti-CMV therapy varies significantly across subgroups

Next, we performed a time-varying Fine–Gray analysis to identify subgroups associated with favorable outcomes when receiving antiviral therapy against CMV. In the overall study cohort, the use of antiviral agents was not linked to ICU mortality (Fig. 3). However, in the subgroup analysis, antiviral therapy was significantly associated with reduced ICU mortality in patients with radiologic findings suggestive of CMV pneumonia, whereas no such association was observed in patients without these findings (*P* for interaction = 0.001). Additionally, antiviral therapy appeared to be associated with increased mortality in patients with positive bacterial cultures (*P* for interaction = 0.002).

## Discussion

We analysed 322 patients admitted to the medical ICU who underwent bronchoscopy for LRT specimen collection within 7 days of admission. The detection of CMV DNA in LRT samples, at levels above the detection threshold (≥ 34.5 IU/mL), was associated with poor outcomes at multiple time points, such as ICU, in-hospital, 30-day, and 90-day mortality. This association was consistently maintained after adjusting for other major clinical factors. Moreover, the subgroups associated with favorable outcomes when receiving antiviral therapy included patients with radiological findings suggestive of CMV pneumonia, while those with co-infection involving other bacterial pathogens appeared to have worse outcomes.

Our study provides important insights into the clinical significance of CMV reactivation in the LRT, which has been underexplored. Although earlier studies have established an association between CMV reactivation and poor outcomes, most have focused detecting CMV in blood plasma [1, 9, 11, 24, 25]. Studies examining CMV DNA in LRT samples have been limited to specific patient groups or univariable analyses, owing to small sample sizes [26, 27]. In this regard, this study highlights CMV reactivation in the LRT as a potential surrogate marker of poor prognosis. Furthermore, our results suggest subgroups that may be associated with favorable or worsened outcomes when receiving antiviral therapy.

CMV reactivation involves both direct and indirect pathways, which contribute to poor outcomes in critically ill patients [28, 29]. Directly, CMV can cause lung tissue damage and lead to severe complications such as ARDS. Indirectly, CMV reactivation triggers the release of pro-inflammatory cytokines, resulting in an exaggerated immune response that may exacerbate underlying conditions and cause multi-organ dysfunction. Although the initial PaO2/FiO2 ratio and the application of mechanical ventilation did not differ between CMV reactivation and non-reactivation groups, the significant difference in ventilator-free days during the first 28 days underscores the persistent effects of CMV-associated inflammatory pathways, potentially contributing to increased mortality. Additionally, co-infections with other pathogens were more frequently observed in the CMV reactivation group, yet antiviral therapy showed limited benefit in the presence of bacterial co-pathogens. This observation suggests that CMV reactivation may reflect underlying critical processes rather than being an isolated driver of poor outcomes. Further research is necessary to elucidate the interplay between CMV reactivation, co-infection, and prognosis.

While the debate over the use of prophylactic or preemptive antiviral therapy in critically ill patients continues [7, 30, 31], our results indicate that anti-CMV therapy may not be universally beneficial. Instead, it appears to be most advantageous for patients with radiological findings suggestive of CMV pneumonia. Conversely, antivirals may be less effective or potentially unfavorable for patients with co-infections involving other bacterial pathogens. Stratification by CMV viral load—whether based on its presence or absence, or using a threshold of 1000 IU/mL—did not reveal a subgroup with improved outcomes when on antiviral therapy. These observations suggest that initiating antiviral therapy should not be guided solely by CMV viral load testing. Rather, it may be more prudent to base decisions on a comprehensive evaluation of radiologic findings and culture results from LRT specimens. Further prospective studies are needed to refine the risk stratification and identify which patients are most likely to benefit from antiviral treatment.

It is noteworthy that 55% of the patients in this study were LRT CMV-negative, underscoring the need to re-evaluate our current practices. Although bronchoscopy is considered safe for collecting samples from the LRT of critically ill patients [14], recent studies have raised concerns regarding its potential complications [32]. The false negative rate (1–sensitivity) of CMV viremia for predicting a positive LRT CMV result was 31%, indicating that plasma CMV testing alone is insufficient to rule out or confirm CMV pneumonia or LRT reactivation. It does not justify bypassing bronchoscopic procedures for CMV testing. Future studies integrating detailed clinical and laboratory data to develop novel methods for identifying patients at a high risk of CMV reactivation and pneumonia could help guide the more selective use of bronchoscopy and avoid unnecessary complications. Encouragingly, LRT CMV positivity showed no difference among patients who underwent bronchial washing, and a sensitivity analysis of this group confirmed the consistency of the main results. This suggests that a smaller lavage volume is adequate for assessing CMV reactivation.

This study has several limitations. This retrospective study is susceptible to selection bias, as patients who did not undergo bronchoscopy or CMV testing using LRT specimen were excluded from the analysis. Therefore, our study should be considered applicable to patients with acute respiratory failure suspected of a LRT infection who undergo bronchoscopy. Second, although CMV reactivation can occur at later stages of ICU stay [10], our analysis was limited to the first bronchoscopic data. Patients who progress in respiratory failure often cannot undergo additional bronchoscopy, while those who recover typically do not require follow-up bronchoscopy. Specimens obtained beyond the early ICU period are also challenging to interpret due to the potential confounding effects of high-dose corticosteroids, frequently administered in cases of acute respiratory failure. To minimize this confounding, we restricted our analysis to specimens collected within the first week of ICU admission. Also, the initiation of antiviral therapy was determined by the attending intensivist, which introduces a potential bias in our analysis, alongside other unmeasured confounders and information inherent to the study design. The findings regarding antiviral treatment should be interpreted with caution. Additionally, we could not clearly define or distinguish isolated CMV reactivation from overt CMV pneumonia, partly due to the lack of histopathological findings and data on extrapulmonary CMV manifestations. Lastly, as the study cohort was drawn from a highly seropositive population [4–6], the applicability of our findings to patient populations with different CMV seroprevalence rates is limited. Additionally, the inclusion of a substantial number of immunocompromised patients with acute respiratory failure limits the generalizability of our findings to the broader population of critically ill patients and those with different risk profiles. However, the study included a relatively large real-world cohort and employed multivariable and subgroup analyses to assess the impact of CMV reactivation in the LRT on prognosis and to identify subgroups that may benefit from antiviral therapy, offering a more nuanced understanding of its effects.

## Conclusion

CMV reactivation in the LRT is associated with poor outcomes in critically ill patients. Anti-CMV therapy was not associated with overall survival outcomes; however, the subgroup with radiologic findings of CMV pneumonia suggested benefits, while the subgroup with bacterial co-infections suggested harmful effects. Randomized controlled trials are needed.

## Availability of data

The dataset used is available from the corresponding author on reasonable request after IRB approval.

## Supplementary Information

Additional file1 (DOCX 15 KB)

## Data Availability

The dataset used is available from the corresponding author on reasonable request after IRB approval.
