# Supplementary material for: Cytomegalovirus reactivation in the lower respiratory tract as an independent risk factor for mortality in critically Ill patients
Source: Crit Care. 2025 May 2;29:177. doi: 10.1186/s13054-025-05324-8 (PMC12048981; doi:10.1186/s13054-025-05324-8)
Supplement: Supplementary file 1 — Additional file1 (DOCX 15 KB) [file 13054_2025_5324_MOESM1_ESM.docx]

| **Supplementary Table 1. Univariable and multivariable subdistribution hazard ratios for ICU mortality using the Fine–Gray model** | | | | |  |
| --- | --- | --- | --- | --- | --- |
|  | | | | |  |
| **Clinical characteristics** | **Univariable analysis** | | **Multivariable analysis** | | |
|  | **SHR (95% CI)** | ***P*** | **adjusted SHR (95% CI)** | ***P*** | |
| LRT CMV positivity | 1.82 (1.23–2.70) | 0.003 | 2.28 (1.46–3.56) | <0.001 | |
| Male sex | 0.85 (0.57–1.28) | 0.44 | 0.88 (0.57–1.36) | 0.55 | |
| Body mass index | 1.06 (1.02–1.10) | 0.002 | 1.09 (1.04–1.14) | <0.001 | |
| APACHE II score | 1.05 (1.03–1.08) | <0.001 | 1.03 (1.003–1.06) | 0.03 | |
| Hematologic malignancy | 1.41 (0.92–2.17) | 0.12 | 1.52 (0.96–2.40) | 0.08 | |
| Lactate level at ICU admission | 1.12 (1.05–1.20) | 0.001 | 1.11 (1.02–1.20) | 0.02 | |
| Lymphopenia | 1.52 (0.87–2.65) | 0.14 | 1.36 (0.74–2.50) | 0.33 | |
| Corticosteroid use | 0.75 (0.46–1.23) | 0.25 | 0.64 (0.38–1.07) | 0.09 | |
| *Aspergillus* positivity | 1.39 (0.92–2.1) | 0.12 | 0.94 (0.59–1.50) | 0.80 | |

SHR; subdistribution hazard ratio; CI, confidence interval; APACHE, Acute Physiology and Chronic Health Evaluation; LRT, lower respiratory tract; CMV, cytomegalovirus; ICU intensive care unit.

| **Supplementary Table 2. Association between LRT CMV positivity and mortality in patients who underwent bronchial washing** | | | | |
| --- | --- | --- | --- | --- |
| **Clinical outcome** | **SHR (95% CI)** | ***P*** | **adjusted SHR (95% CI)** | ***P*** |
| ICU mortality | 2.10 (1.28–3.45) | 0.003 | 2.41 (1.36–4.28) | 0.003 |
| In-hospital mortality | 2.32 (1.59–3.39) | <0.001 | 2.90 (1.85–4.54) | <0.001 |
| 30-day mortality | 2.42 (1.57–3.72) | 0.001 | 3.08 (1.84–5.14) | <0.001 |
| 90-day mortality | 2.39 (1.61–3.55) | <0.001 | 3.05 (1.91–4.88) | <0.001 |
| LRT, lower respiratory tract; CMV, cytomegalovirus; SHR; subdistribution hazard ratio; CI, confidence interval; ICU, intensive care unit.  Multivariable analyses adjusted for sex, body mass index, APACHE II score, hematologic malignancy, initial lactate levels, lymphopenia, corticosteroid use, and *Aspergillus* positivity in LRT specimen. | | | | |
